# OTI1H1-Defined MAGEA3-Directed Immunoreactivity and Survival in Pancreatic Ductal Adenocarcinoma: A Single-Center Study

**DOI:** 10.3390/jcm15218276

**Published:** 2026-10-24

**Authors:** Goran Glavčić, Marin Glavčić, Suzana Janković, Dubravka Mužina, Zdenko Bilić, Nina Blažević, Zvonimir Misir, Jasmina Hranjec, Andro Košec, Petra Radulović

**Affiliations:** 1 Clinic for Surgery, University Hospital Centre “Sestre milosrdnice”, 10000 Zagreb, Croatia; suzana.jankovic@kbcsm.hr (S.J.); dubravka.muzina@kbcsm.hr (D.M.); zdenko.bilic@kbcsm.hr (Z.B.); zvonimir.misir@kbcsm.hr (Z.M.); jasmina.hranjec@kbcsm.hr (J.H.); 2 School of Medicine, Catholic University of Croatia, 10000 Zagreb, Croatia; 3 Clinic for Orthopedics and Traumatology, University Hospital Dubrava, 10000 Zagreb, Croatia; mglavcic@kbd.hr; 4 School of Dental Medicine, University of Zagreb, 10000 Zagreb, Croatia; petra.radulovic@kbcsm.hr; 5 Clinic for Internal Medicine, University Hospital Centre “Sestre milosrdnice”, 10000 Zagreb, Croatia; nina.blazevic@kbcsm.hr; 6 Department of Otorhinolaryngology and Head and Neck Surgery, University Hospital Centre “Sestre milosrdnice”, 10000 Zagreb, Croatia; andro.kosec@kbcsm.hr; 7 School of Medicine, University of Zagreb, 10000 Zagreb, Croatia; 8 Clinical Department of Pathology and Cytology “Ljudevit Jurak”, University Hospital Centre “Sestre milosrdnice”, 10000 Zagreb, Croatia

**Keywords:** pancreatic ductal adenocarcinoma, MAGEA3, OTI1H1, immunohistochemistry, overall survival

## Abstract

**Background/Objectives:** Melanoma-associated antigen A3 (MAGEA3) is a cancer–testis antigen with potential biological and prognostic relevance in pancreatic ductal adenocarcinoma (PDAC). This study evaluated the association between OTI1H1-defined MAGEA3-directed immunoreactivity and clinicopathological characteristics and overall survival after pancreatic resection for PDAC. **Methods:** This retrospective cohort included 76 patients with histologically confirmed PDAC. Patients who died within 30 postoperative days were excluded, and all tumors were retrospectively restaged according to the eighth edition of the AJCC TNM classification. Immunohistochemical staining was assessed using the mouse monoclonal anti-MAGE3 antibody clone OTI1H1 (ab140678, Abcam) by a single experienced pathologist blinded to clinical and outcome data. The percentage of immunoreactive tumor cells was retained as the principal exploratory quantitative measure; the prespecified <10% versus ≥10% classification was evaluated secondarily. **Results:** Thirty-six of 76 tumors (47.4%) had ≥10% OTI1H1-reactive tumor cells. When staining was modeled continuously, the adjusted HR per 10-percentage-point increase was 0.969 (95% CI 0.898–1.046; *p* = 0.420). For the secondary binary classification, the adjusted HR for ≥10% versus <10% staining was 0.745 (95% CI 0.417–1.331; *p* = 0.320). No statistically clear association with overall survival was observed; however, the confidence intervals do not exclude potentially clinically relevant associations in either direction. **Conclusions:** No statistically clear association between OTI1H1-defined immunoreactivity and overall survival was observed in this cohort. Because OTI1H1 has known cross-reactivity with selected MAGE-A family proteins and no orthogonal molecular validation was performed, these findings should be interpreted as assay-specific and do not establish biological inactivity of MAGEA3 or related MAGE-A proteins.

## 1. Introduction

### 1.1. Clinical Context

Pancreatic ductal adenocarcinoma (PDAC) is the predominant histological subtype of pancreatic cancer and remains a major cause of cancer-related mortality [1,2]. Surgical resection is the only potentially curative treatment, but only a minority of patients present with resectable disease, and long-term survival remains limited even after curative-intent surgery [3,4,5]. Pathological T and N categories, resection-margin status, and tumor differentiation remain important determinants of outcome [6,7].

### 1.2. MAGE Proteins

The melanoma-associated antigen (MAGE) family comprises more than 40 proteins. Type I MAGE proteins, including the MAGE-A, -B, and -C subfamilies, are cancer–testis antigens whose physiological expression is largely restricted to germline tissues but is aberrantly activated in multiple malignancies [8,9,10]. Several MAGE proteins have been implicated in tumor-cell survival, DNA-damage repair, and treatment resistance, and their expression has been associated with adverse clinicopathological features in some cancers [11,12,13,14,15,16,17,18,19].

The relatively restricted distribution of MAGE antigens in normal tissues and their immunogenicity have stimulated interest in therapeutic targeting. Nevertheless, clinical studies of MAGE-based vaccines and related approaches have produced inconsistent results and have not consistently demonstrated a survival benefit [20,21,22].

### 1.3. MAGEA3 in Pancreatic Cancer

MAGEA3 is predominantly localized in the cytoplasm and can be recognized by cytotoxic T lymphocytes [23,24]. Preclinical studies suggest that MAGEA3 may promote tumor-cell survival and may be immunologically targetable in pancreatic cancer [25,26]. However, clinical evidence remains limited.

In the principal clinical study of pancreatic cancer, MAGEA3 expression was detected in 44% of tumors and was associated with poorer survival in unadjusted analyses; however, it was not an independent predictor after adjustment for clinicopathological variables [27]. This discrepancy between biological rationale and limited clinical prognostic evidence supports further evaluation in additional clinical datasets. Contemporary PDAC research has also examined other immune-related tissue and molecular biomarkers, including PD-L1 expression and immune-subtype classifiers, illustrating the broader effort to identify reproducible prognostic markers in pancreatic cancer [28,29].

### 1.4. Study Aim

This study aimed to evaluate the prognostic association of OTI1H1-defined MAGEA3-directed immunoreactivity in surgically resected PDAC by assessing its relationship with pathological T category, pathological nodal status, other clinicopathological characteristics, and overall survival. The original hypothesis was that greater tumor-cell immunoreactivity would be associated with more advanced pathological disease and poorer clinical outcome. The study was not designed to establish treatment-predictive value, target-specific molecular activity, or biological inactivity of MAGEA3 or related MAGE-A proteins.

## 2. Materials and Methods

### 2.1. Samples

Archival formalin-fixed, paraffin-embedded tissue specimens were obtained from patients who underwent pancreatic resection for histologically confirmed PDAC at University Hospital Centre “Sestre milosrdnice” between 1 January 2003 and 31 December 2019. Inclusion required complete clinical and follow-up information, adequate histopathological material for immunohistochemical assessment, and absence of another malignant tumor. Patients who died within 30 postoperative days were excluded. The final MAGEA3-directed analytic cohort comprised 76 patients. The present study and the previously published analysis of CD86/CD163-positive intratumoral macrophages [30] were derived from the same broader archival source population of 136 resected PDAC cases; however, the final 76-patient analytic cohorts were not identical because adequate archival tissue and complete clinical/follow-up documentation were not available for all patients for both analyses. The exact degree of overlap was not prospectively recorded. Because a complete contemporaneous screening log was not retained in a form permitting reliable case-by-case reconstruction, the number of exclusions attributable to each individual reason cannot be reported reliably.

Clinical and surgical data were obtained from the institutional Hospital Information System, and dates of death were obtained from the Croatian National Cancer Registry. All tumors from the 2003–2019 study period were retrospectively restaged according to the eighth edition of the AJCC TNM classification to ensure consistent pathological T and N categorization [3,6]. A positive resection margin (R1) was defined as microscopic tumor involvement at the inked resection margin. Vital status and follow-up were updated to ensure at least 24 months of potential follow-up for patients operated at the end of the inclusion period. Overall survival was calculated from the date of surgery to death from any cause or the last documented follow-up, with surviving patients censored at their last documented follow-up. Potential follow-up was additionally summarized using the reverse Kaplan–Meier method.

Initiation of postoperative adjuvant therapy was not an eligibility criterion. None of the included patients received neoadjuvant therapy, and all patients in the final cohort initiated postoperative adjuvant therapy in accordance with the oncological guidelines and clinical practice applicable at the time of treatment. However, complete information regarding treatment completion, regimen, dose intensity, and duration was not consistently available.

All data were anonymized before analysis. The study was conducted in accordance with the Declaration of Helsinki and approved by the Ethics Committee of University Hospital Centre “Sestre milosrdnice”, Zagreb, Croatia (approval No. 251-29-11-21-01-13; approval date: 8 April 2021).

### 2.2. Immunohistochemistry

Tissue samples were fixed in 10% buffered formalin, embedded in paraffin, and sectioned at 5 µm. After deparaffinization, routine hematoxylin–eosin staining was performed for histopathological assessment. For immunohistochemistry, deparaffinization and pretreatment were performed using the Dako PT Link protocol with high-pH buffer (pH 9) at 95 °C for 30 min. Immunohistochemical staining was performed on an Autostainer Link 48 (Dako) using the mouse monoclonal anti-MAGE3 antibody clone OTI1H1 (ab140678, Abcam, UK) at a dilution of 1:500. Documented automated staining steps included peroxidase blocking for 5 min, horseradish-peroxidase (HRP) polymer incubation for 20 min, primary-antibody incubation for 30 min, 3,3′-diaminobenzidine (DAB) chromogen development for 5 min, and hematoxylin counterstaining for 5 min. Colon tissue was used as the positive control. The antibody was generated against recombinant full-length human MAGEA3; according to the manufacturer’s current product information, OTI1H1 cross-reacts with MAGEA2, MAGEA6, MAGEA8, and MAGEA12 and does not cross-react with MAGEA1 or MAGEA4 [31]. The retained protocol documentation records colon tissue as the positive control but does not include control-run results or a separate negative reagent control.

One representative tumor slide per patient was assessed. The percentage of tumor cells showing cytoplasmic immunoreactivity in the epithelial tumor component was estimated across the whole selected slide. All immunohistochemical slides were evaluated by a single experienced pathologist (P.R.), who was blinded to the patients’ clinical characteristics and outcome data. The retained protocol documentation does not specify a formal rule for selection of the representative tumor slide. Formal inter-observer and intra-observer reproducibility testing, including blinded repeat scoring, was not performed.

The 10% threshold was prespecified during the original pathological assessment as a pragmatic operational boundary. Tumors with staining in <10% of tumor cells were classified as negative and those with staining in ≥10% as positive; this rule was intended to prevent minimal weak/focal signal and background staining from defining the positive category. The threshold was not derived by outcome optimization, ROC/Youden analysis, or a biologically validated breakpoint and is not presented as a PDAC-specific prognostic threshold. Percentage-based approaches have been used in prior studies of MAGEA3 and other cancer–testis antigens [32,33,34], although those studies differed in tumor type and assay methodology and therefore do not validate the exact 10% OTI1H1 threshold used here. Accordingly, the raw staining percentage was retained as the principal exploratory quantitative measure, whereas the <10% versus ≥10% classification was used as a secondary operational analysis. An H-score was not prespecified in the original assessment, and staining intensity, spatial distribution, and intratumoral heterogeneity were not systematically recorded.

### 2.3. Statistical Analysis

Continuous variables are presented as mean ± standard deviation or median and range/interquartile range, as appropriate, and categorical variables as counts and percentages. Normality was assessed using the Kolmogorov–Smirnov test. Associations between demographic, clinical, histopathological, and immunohistochemical variables were examined using appropriate parametric or non-parametric tests and Spearman’s rho correlation coefficient. The raw percentage of OTI1H1-reactive tumor cells was treated as the principal exploratory quantitative exposure, while the prespecified <10% versus ≥10% classification was evaluated secondarily. Baseline characteristics were also summarized descriptively by staining group. There were no missing values for the variables used in the primary survival models.

Overall survival was defined as the interval from surgery to death from any cause or the last documented follow-up, with patients alive at last follow-up treated as censored observations. Survival was estimated using the Kaplan–Meier method, and potential follow-up was estimated using the reverse Kaplan–Meier method. Cox proportional-hazards regression was used to evaluate prognostic associations. Candidate covariates were selected on clinical grounds rather than by univariable *p*-value screening. The adjusted binary model included OTI1H1 staining status (≥10% vs. <10% [reference]), age (per 10 years), sex (male vs. female [reference]), pathological T category (pT2 and pT3 vs. pT1 [reference]), pathological nodal status (pN-positive [pN1/pN2] vs. pN0 [reference]), resection-margin status (R1 vs. R0 [reference]), and calendar year of surgery (per 5 years). Because only three patients had pN2 disease, pN1 and pN2 were combined for the adjusted models. A parallel continuous model used the same adjustment set and modeled staining per 10-percentage-point increase. The Efron method was used for tied event times. The proportional-hazards assumption was assessed using Schoenfeld residuals tested for correlation with log-transformed follow-up time. For continuous staining percentage, age, and calendar year, linear specifications were compared with natural-cubic-spline forms using likelihood-ratio tests. Postoperative complications and reoperation were not included as Cox-model covariates because they occurred after the survival time origin and their occurrence times were not encoded for time-dependent modeling.

The exploratory 20% cut-off sensitivity analysis reclassified only one of the 76 tumors and was retained only as a brief robustness check; it was not interpreted as validation of the prespecified 10% threshold. All tests were two-sided, and *p*-values ≤ 0.05 were considered statistically significant. Statistical analyses were performed using MedCalc software (version 11.2.1; MedCalc Software Ltd., Ostend, Belgium) and IBM SPSS Statistics for Windows, version 22.0 (IBM Corp., Armonk, NY, USA).

## 3. Results

### 3.1. Patient Characteristics

The cohort included 76 patients who underwent pancreatic resection for histologically confirmed PDAC. There were 32 men and 44 women. The mean age was 65.1 ± 10.0 years, and the median age was 66.5 years. Thirty-six tumors (47.4%) had ≥10% OTI1H1-reactive tumor cells and 40 (52.6%) had <10%. The mean percentage of OTI1H1-reactive tumor cells was 32.3%, with a median of 5%, an interquartile range of 0–80%, and a range of 0–95%. Baseline demographic, clinicopathological, surgical, and outcome characteristics for the total cohort and by staining group are summarized in Table 1. No variables used in the primary survival models were missing.

### 3.2. Follow-Up Duration and Survival Analysis

The mean observed follow-up (time-to-event) was 26.0 ± 22.2 months, and the median was 19.33 months (range, 1.77–128.5 months). The final verified dataset contained 63 deaths and 13 censored observations. The 13 censored patients had 30.17–128.5 months of observed follow-up. Median potential follow-up estimated using the reverse Kaplan–Meier method was 56.37 months.

**Figure 1:**
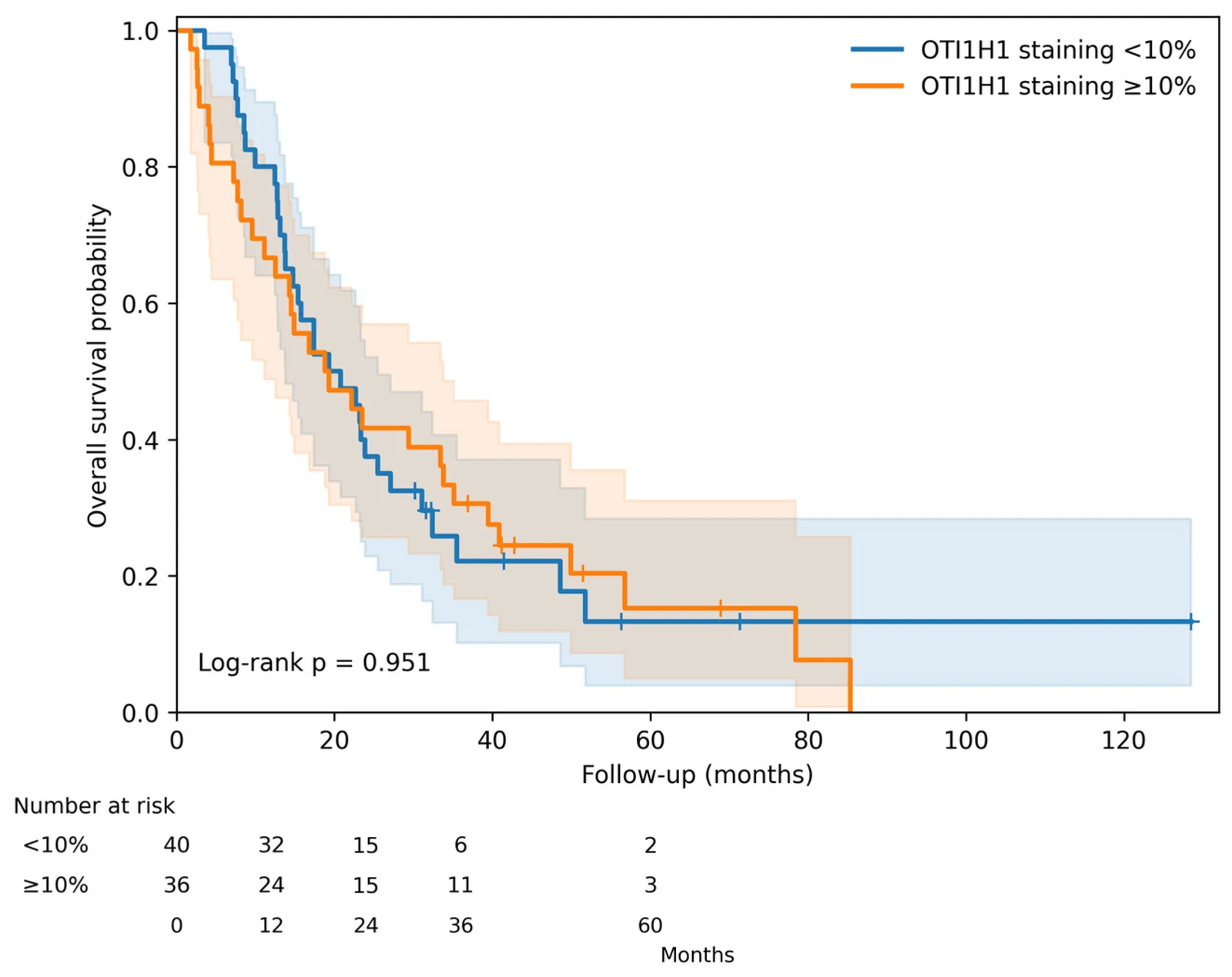
Kaplan–Meier overall survival according to the prespecified <10% versus ≥10% OTI1H1 staining classification. Shaded bands indicate 95% confidence intervals; plus signs indicate censored observations. Numbers at risk are shown below the plot. Log-rank *p* = 0.951.

Overall survival stratified by the prespecified <10% versus ≥10% OTI1H1 staining classification is shown in Figure 1, including 95% confidence bands, censoring marks, and numbers at risk.

**Figure 2:**
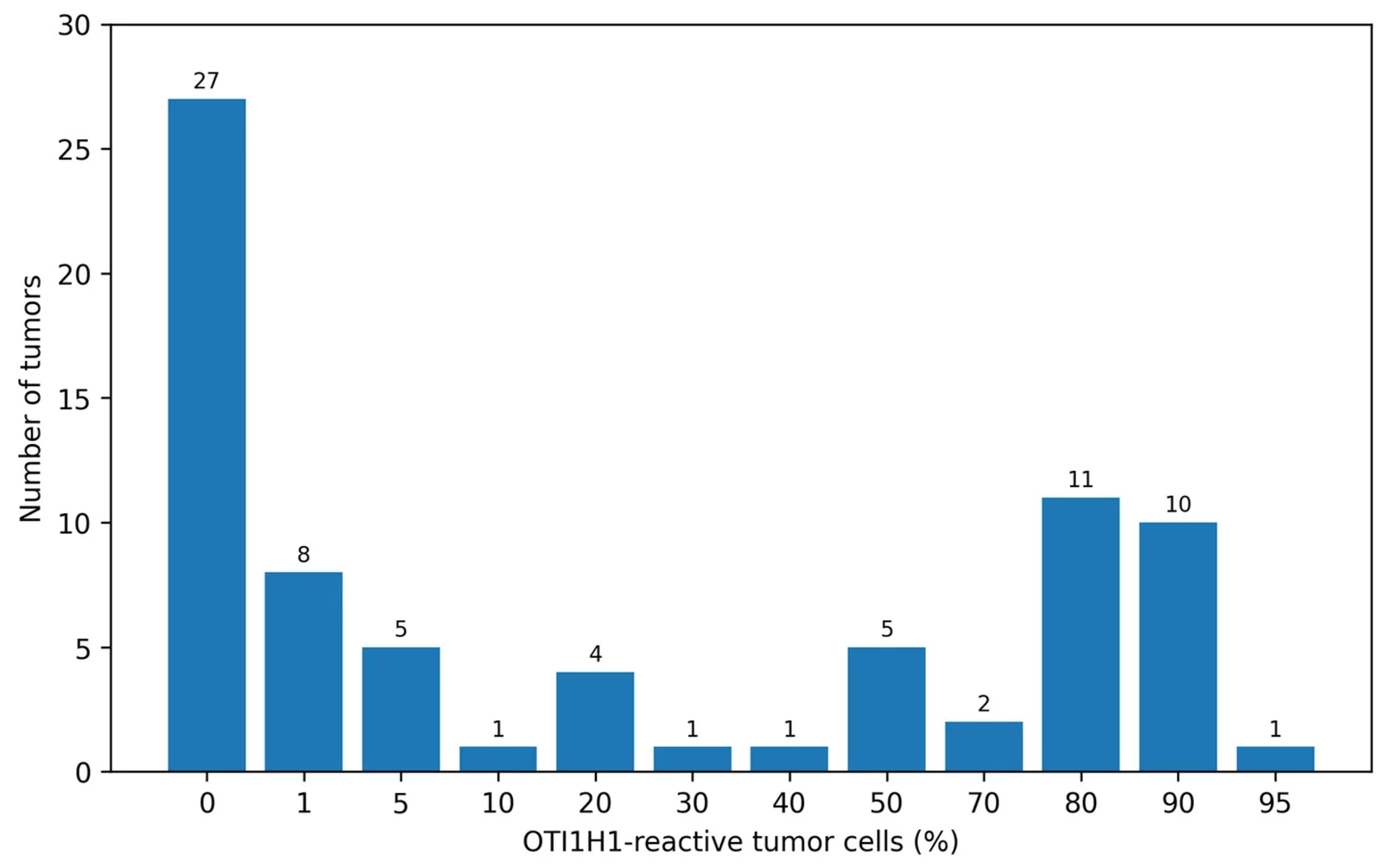
Distribution of the percentage of OTI1H1-reactive tumor cells across the 76 PDAC specimens. The mean was 32.3%, the median was 5%, the interquartile range was 0–80%, and the observed range was 0–95%.

The distribution of the raw percentage of OTI1H1-reactive tumor cells is shown in Figure 2.

Median overall survival was 19.33 months in the ≥10% staining group and 20.77 months in the <10% group (log-rank *p* = 0.951). In the principal exploratory continuous analysis, the adjusted HR per 10-percentage-point increase in OTI1H1-reactive tumor cells was 0.969 (95% CI 0.898–1.046; *p* = 0.420). In the secondary binary analysis, the univariable HR for ≥10% versus <10% staining was 1.015 (95% CI 0.616–1.672; *p* = 0.953), and the adjusted HR was 0.745 (95% CI 0.417–1.331; *p* = 0.320). No statistically clear association with overall survival was observed; however, the confidence intervals do not exclude potentially clinically relevant associations in either direction. Complete univariable and adjusted Cox model results are shown in Table 2.

Proportional-hazards diagnostics showed no statistically significant time dependence for any covariate in either retained adjusted model (all *p* > 0.05); for the staining variable, *p* = 0.075 in the binary model and *p* = 0.144 in the continuous model. Flexible natural-cubic-spline specifications did not improve model fit for continuous staining percentage (*p* > 0.99), age (*p* = 0.272), or calendar year (*p* = 0.775), supporting the linear specifications used in the reported models.

### 3.3. Associations Between Demographic, Clinical, and Histopathological Variables

Associations between OTI1H1-defined immunoreactivity and demographic, clinical, and histopathological variables were evaluated using both the raw percentage of reactive tumor cells and the prespecified binary ≥10% classification.

Neither representation demonstrated a statistically clear association with age, sex, pathological T category, pathological nodal status, resection-margin status, postoperative complications, reoperation, or type of surgery in this cohort.

**Figure 3:**
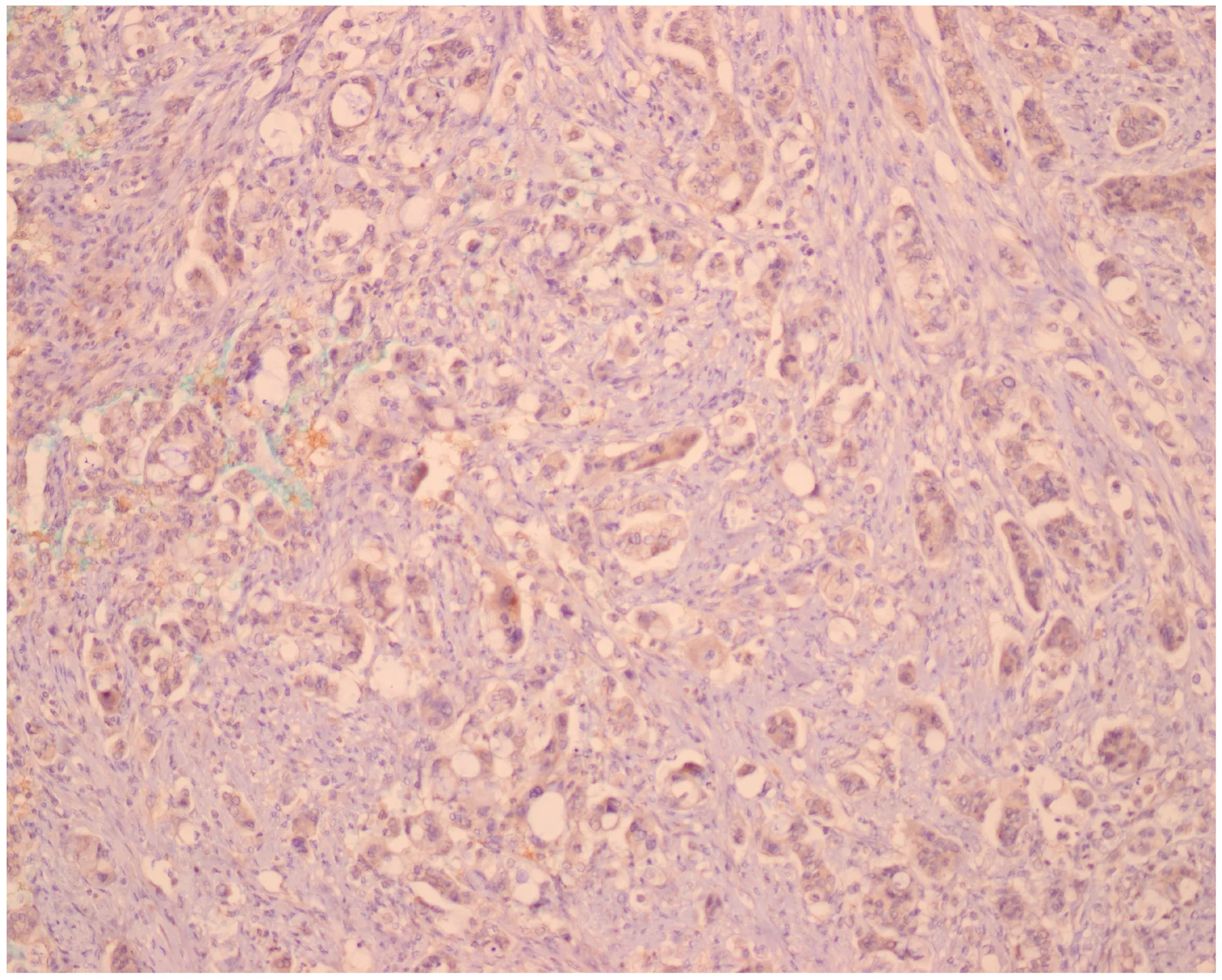
Representative immunohistochemical micrograph showing OTI1H1-defined MAGEA3-directed immunoreactivity in pancreatic ductal adenocarcinoma cells. Immunoreactivity is predominantly cytoplasmic and of weak-to-moderate intensity. Original magnification ×200.

**Figure 4:**
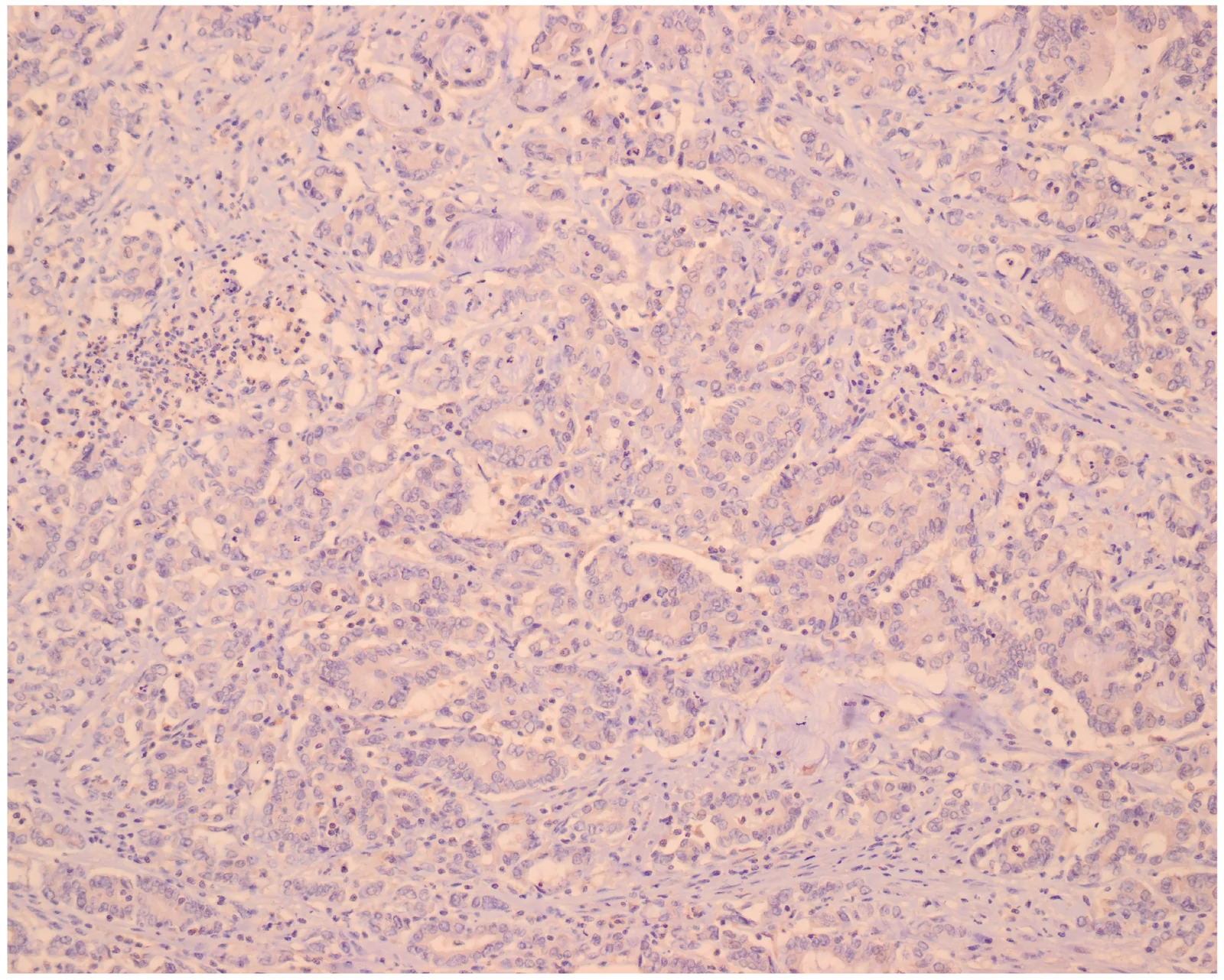
Representative immunohistochemical micrograph of pancreatic ductal adenocarcinoma showing negative OTI1H1 immunoreactivity in tumor cells. This image represents a negatively stained tumor specimen and not a reagent-negative control. Original magnification ×200.

The exploratory 20% cut-off sensitivity analysis reclassified only one of the 76 tumors and did not materially change the survival conclusion; it is therefore reported only as a limited robustness check and not as evidence validating the 10% threshold. Representative PDAC specimens with positive and negative tumor-cell OTI1H1 immunoreactivity are shown in Figure 3 and Figure 4, respectively. The displayed micrographs are original high-resolution images from specimens included in the present study.

## 4. Discussion

In this retrospective single-center cohort of 76 surgically treated patients with PDAC, 36 tumors (47.4%) showed ≥10% OTI1H1-reactive tumor cells using the prespecified operational classification. Neither the principal exploratory continuous staining percentage nor the secondary binary classification showed a statistically clear association with pathological T category, pathological nodal status, other examined clinicopathological variables, or overall survival. The adjusted HR was 0.969 per 10-percentage-point increase in staining (95% CI 0.898–1.046) and 0.745 for the ≥10% versus <10% classification (95% CI 0.417–1.331). These findings should be interpreted as an absence of a statistically clear prognostic association in this cohort; the confidence intervals remain compatible with potentially clinically relevant associations in either direction.

The findings should be considered in the context of the earlier study by Kim et al., which detected MAGEA3 expression in 44% of pancreatic tumors and reported an association with survival in unadjusted analysis but not after multivariable adjustment [27]. Direct comparison is limited by substantial methodological and clinical heterogeneity. Kim et al. primarily evaluated MAGEA3 expression by quantitative real-time RT-PCR and used immunohistochemistry as confirmatory protein assessment, whereas the present study evaluates percentage-based OTI1H1 immunoreactivity. Differences in antibody reagents and assay specificity, patient characteristics, disease-stage distribution, treatment era and exposure, and multivariable covariate structure may all contribute to differences between unadjusted and adjusted prognostic signals. Accordingly, the present work should be viewed as an additional assay-specific clinical assessment rather than as independent validation of a target-specific prognostic biomarker. The previously published macrophage study [30] arose from the same broader archival source population but used a partially overlapping, non-identical final analytic cohort.

Importantly, the absence of a statistically clear prognostic association for the OTI1H1-defined immunohistochemical signal does not imply that MAGEA3 or related MAGE-A proteins lack biological relevance in PDAC. Experimental studies have reported pro-survival effects and enhanced immune recognition under selected conditions [25,26]. The present study did not measure transcriptional activity, protein interactions, pathway activation, immune recognition, or treatment response and therefore cannot establish biological inactivity, predictive value, or therapeutic efficacy.

The 10% cut-off was a prespecified pragmatic operational criterion used during the original pathological assessment, not an outcome-derived or biologically validated breakpoint. Prior MAGEA3 and cancer–testis-antigen studies have used percentage-based approaches [32,33,34], but differences in tumor type, antibody reagents, and scoring methods prevent those studies from validating the exact 10% OTI1H1 threshold used in PDAC here. Percentage-only scoring also does not capture staining intensity, spatial distribution, or intratumoral heterogeneity. Because these features were not systematically recorded in the original assessment, retrospective creation of an H-score would require a new slide review and would introduce a post hoc measurement strategy. The raw staining percentage was therefore retained as the principal exploratory representation, with the binary classification analyzed secondarily.

The change in the staining HR from 1.015 in the univariable binary model to 0.745 after adjustment appears to reflect the distribution of clinical covariates rather than a change in event data. Compared with the <10% group, the ≥10% group was treated earlier on average (mean calendar year 2013.6 vs. 2015.4), had a higher proportion of pT3 tumors (50.0% vs. 35.0%), and had more R1 resections (30.6% vs. 17.5%). Sequential adjustment moved the staining HR toward 0.745, with the largest additional shift occurring after inclusion of calendar year. This adjusted estimate should therefore be interpreted as a confounding-adjusted association and not as evidence of a protective biological effect.

The study has several limitations. The cohort was relatively small, retrospective, and derived from a single center. All slides were evaluated by one experienced pathologist; independent second-reader assessment and formal inter-observer or intra-observer reproducibility testing, including blinded repeat scoring, were not performed. Visual percentage estimates close to the 10% boundary may therefore be sensitive to repeat-read variation. In addition, OTI1H1 is not MAGEA3-exclusive: the manufacturer reports cross-reactivity with MAGEA2, MAGEA6, MAGEA8, and MAGEA12 [31]. Without confirmation by an independent antibody or an orthogonal molecular method such as quantitative PCR or Western blotting, the observed signal cannot be interpreted as exclusive molecular quantification of MAGEA3. Although the main historical staining protocol could be reconstructed, the retained protocol documentation does not include control-run results, a separate negative reagent control, or a formal rule for selecting the representative tumor slide. Original high-resolution micrographs were available, but the archived image files did not retain calibration metadata sufficient to generate a reliable micrometer scale bar retrospectively; matched H&E images and archived run-specific control images were not available for presentation. These limitations reinforce the assay-specific and exploratory interpretation of the findings.

Additional limitations arise from the prolonged inclusion period from 2003 to 2019. Although all tumors were retrospectively restaged according to AJCC eighth-edition criteria, changes in surgical technique, perioperative care, and adjuvant treatment may have introduced temporal and treatment-related confounding; calendar year was therefore included in the adjusted models. None of the patients received neoadjuvant therapy, and all patients in the final cohort initiated postoperative adjuvant therapy, but treatment completion, exact regimen, dose intensity, and duration could not be reconstructed reliably. Tumor grade and additional potentially relevant pathological variables such as lymphovascular and perineural invasion were not available in the final analytic dataset in a form permitting reliable case-level reconstruction. Genomic data, including KRAS, TP53, and homologous-recombination-deficiency-related genes, were also unavailable. Finally, a complete contemporaneous screening log was not retained, so exclusion counts by individual reason cannot be reconstructed reliably.

Future studies should use multicenter cohorts, standardized and fully documented quantitative immunohistochemical protocols, independent pathology review, and orthogonal molecular validation. Such studies should also integrate genomic characterization, detailed treatment exposure, and clinically appropriate measures of incremental prognostic performance before any claim of clinical utility or treatment-predictive value is made.

## 5. Conclusions

In this retrospective cohort, no statistically clear association was observed between OTI1H1-defined MAGEA3-directed immunoreactivity and pathological T category, pathological nodal status, or overall survival. The confidence intervals do not exclude potentially clinically relevant associations in either direction. Because OTI1H1 has known cross-reactivity with selected MAGE-A family proteins and no orthogonal molecular validation was performed, the findings are assay-specific and should not be interpreted as exclusive target-specific quantification or as evidence against a biological role of MAGEA3 or related MAGE-A proteins. Larger multicenter studies incorporating standardized and fully documented quantitative scoring, independent pathological assessment, orthogonal molecular validation, genomic characterization, and detailed treatment data are required.

## Figures and Tables

**Table 1:** Baseline Patient Demographics, Clinicopathological Characteristics, Outcomes, and OTI1H1-Defined Staining Groups.

Variable	Total Cohort (*n* = 76)	<10% Staining (*n* = 40)	≥10% Staining (*n* = 36)
Age, mean ± SD (years)	65.1 ± 10.0	65.35 ± 9.51	64.92 ± 10.71
Sex, male/female	32/44	18/22	14/22
pT1/pT2/pT3	11/33/32	5/21/14	6/12/18
pN0/pN1/pN2	38/35/3	19/18/3	19/17/0
R1 resection margin, *n*	18	7	11
Pancreaticoduodenectomy (Whipple), *n*	56	28	28
Total pancreatectomy, *n*	8	7	1
Subtotal pancreatectomy, *n*	3	2	1
Distal pancreatectomy, *n*	9	3	6
Postoperative complications, *n*	13	6	7
Reoperation, *n*	11	6	5
Calendar year of surgery, mean	2014.5	2015.4	2013.6
Deaths/censored observations	63/13	32/8	31/5

Note: R1 was defined as microscopic tumor involvement at the inked resection margin. No values were missing for the variables shown.

**Table 2:** Univariable and Multivariable Cox Proportional-Hazards Models for Overall Survival.

Covariate	Univariable HR (95% CI); *p*	Adjusted Binary Model HR (95% CI); *p*	Adjusted Continuous Model HR (95% CI); *p*
OTI1H1 staining ≥10% vs. <10%	1.015 (0.616–1.672); 0.953	0.745 (0.417–1.331); 0.320	—
OTI1H1 staining, per +10 percentage points	1.016 (0.952–1.084); 0.639	—	0.969 (0.898–1.046); 0.420
Age, per 10 years	0.990 (0.745–1.315); 0.943	1.184 (0.832–1.684); 0.348	1.189 (0.835–1.693); 0.338
Male vs. female	1.410 (0.852–2.335); 0.181	1.563 (0.910–2.685); 0.105	1.577 (0.915–2.719); 0.101
pT2 vs. pT1	1.242 (0.578–2.669); 0.579	1.275 (0.533–3.047); 0.585	1.317 (0.554–3.130); 0.534
pT3 vs. pT1	1.936 (0.912–4.113); 0.086	1.839 (0.798–4.235); 0.152	1.871 (0.812–4.309); 0.141
pN-positive vs. pN0	1.764 (1.056–2.946); 0.030	1.667 (0.963–2.886); 0.068	1.663 (0.960–2.880); 0.070
R1 vs. R0	1.874 (1.078–3.258); 0.026	2.146 (1.170–3.933); 0.014	2.128 (1.159–3.905); 0.015
Calendar year, per 5 years	0.648 (0.479–0.877); 0.005	0.589 (0.404–0.860); 0.006	0.589 (0.402–0.863); 0.007

Note: *n* = 76 and 63 deaths for all retained models. Reference categories are <10% staining, female sex, pT1, pN0, and R0. pN-positive combines pN1 and pN2 because only three patients were pN2. The adjusted continuous model used the same covariates as the adjusted binary model. HR, hazard ratio; CI, confidence interval.

## Data Availability

The data presented in this study are available from the corresponding author upon reasonable request, subject to institutional and privacy restrictions.

## Abbreviations

The following abbreviations are used in this manuscript:

**Abbreviation**	**Definition**
MAGE	melanoma-associated antigen
MAGEA3	melanoma-associated antigen A3
PDAC	pancreatic ductal adenocarcinoma
